# Humoral Influence of Repeated Lineage-Negative Stem/Progenitor Cell Administration on Articulatory Functions in ALS Patients

**DOI:** 10.1155/2020/8888271

**Published:** 2020-12-14

**Authors:** Anna Sobuś, Bartłomiej Baumert, Wioletta Pawlukowska, Monika Gołąb-Janowska, Edyta Paczkowska, Agnieszka Wełnicka, Agnieszka Meller, Karolina Machowska-Sempruch, Alicja Zawiślak, Karolina Łuczkowska, Sławomir Milczarek, Bogumiła Osękowska, Krzysztof Safranow, Iwona Rotter, Przemysław Nowacki, Bogusław Machaliński

**Affiliations:** ^1^Department of General Pathology, Pomeranian Medical University, Szczecin, Poland; ^2^Department of Medical Rehabilitation and Clinical Physiotherapy, Pomeranian Medical University, Szczecin, Poland; ^3^Department of Neurology, Pomeranian Medical University, Szczecin, Poland; ^4^Department of Biochemistry and Medical Chemistry, Pomeranian Medical University, Szczecin, Poland

## Abstract

Amyotrophic lateral sclerosis (ALS) remains a fatal, neurodegenerative disease frequently leading to dysarthria and impaired swallowing. Better understanding of ALS pathophysiology is prompting the use of humoral cell therapies. Hence, a repeated cellular therapy was applied to ALS patients as an attempt to prevent speech deterioration. Autologous bone marrow-derived lineage-negative (Lin^−^) cells were intrathecally administered three times at six-week intervals to 42 sporadic ALS patients. Patients were examined for articulatory functions using subjective (VHI) and objective (FDA) scales. Selected trophic, proinflammatory factors and expression profiles of miRNA were measured in cerebrospinal fluid (CSF) and plasma by multiplex Luminex and q-PCR in different timepoints. Of the 42 patients who received the Lin^−^ cells, 6 showed improvement in articulatory functions, 27 remained stable, and 9 deteriorated after 18 weeks of therapy according to FDA scale. Clinical improvement was particularly evident by the 7^th^ day of each cell application and concerned better cough and swallow reflex, soft palate, laryngeal time, pitch, and volume. These results correlated with significant changes in the concentration of various trophic and proinflammatory factors and miRNA expression profiles. A multiple application of Lin^−^ cells proved to be safe and feasible. The repeated procedure can potentate a humoral effect and prevent speech deterioration. A short-lasting trophic effect of each Lin^−^ cells administration was observed on local and systemic level. However, further in-depth studies are necessary to sustain the beneficial effect.

## 1. Introduction

Amyotrophic lateral sclerosis (ALS) is a progressive motor neuron disease that leads to a decline in motor function, including articulatory deterioration. Speech impairment, occurring in 80-95% of patients, begins with a decrease in the range and slowing of oral movements and then eventually leads to anarthria [1, 2]. Speech disorders arise from bulbar involvement, leading to changes in four areas: articulation, respiration, phonation, and resonance [3]. It has been shown that speech deterioration associated with the length of phonation and voice loudness is more dynamic in men [4]. Changes occurring in these areas have serious consequences on the functioning of patients with ALS. Previous studies confirmed that speech disorders have a direct, negative impact on the quality of life [5]. Impairment of articulatory, phonation, and resonance organs not only leads to speech disorders in the form of dysarthria but also affects swallowing causing dysphagia [6]. In the initial phase of ALS with bulbar involvement, patients have problem with the food bolus formation; then increasing restrictions on the mobility of articulatory organs lead to difficulties in swallowing fluids. Finally, patients need to be provided with enteral nutrition to sustain alimentation [7]. Progressive bulbar dysfunction in ALS patients also leads to recurrent aspiration, obstruction, and infection of the lower respiratory tract [8]. Respiratory disorders cause shortness of breath and nocturnal hyperventilation, which may contribute to subsequent excessive drowsiness and fatigue during day [9]. Most of the patients with ALS require mechanical ventilation shortly after the onset of respiratory disorders [10]. Acute or acute-on-chronic respiratory failure is the main cause of death in ALS patients [7]. Assessment of progression of articulatory, phonation, and respiratory disorders remains a very important clinical challenge. There are only few effective tools able to assess promptly the disease process and without the need for frequent visits to medical facilities. Based on our research, it appears that Voice Handicap Index (VHI) is the best screening tool showing a high correlation with objective scales standardly used in clinics (i.e., Frenchay Dysarthria Assessment (FDA)) [11]. Despite its undeniable advantages, VHI remains only a supplementary subjective tool in articulation assessment, and for objective clinical evaluation, the specialized FDA scale should be applied by an experienced speech therapist.

Up to date, there is no effective treatment for ALS. The only available drugs (riluzole and edaravone) prolong the survival by approximately 2-3 months and do not act with the same efficacy in all patients [12]. Therefore, the search for a new therapy has turned towards other directions including stem/progenitor cells used as a potential source of trophic support for degenerating neurons. Various populations of stem cells have been previously reported to secrete trophic factors in the central nervous system (CNS) [13]. One of them is a population of lineage-negative cells which are highly enriched in CD34^+^ and CD133^+^ and express markers involved in stem/progenitor cells (SPCs) migration, adhesion, and homing to the bone marrow [14]. Moreover, they express neurotrophic factors, which could beneficially influence the degenerating neurons and neural synapses in neurodegenerative disorders, in higher levels than other nucleated cell types [14]. Our studies utilizing this cell population have proved that they constitute a safe and feasible route of trophic support not only when administered intrathecally but also into the coronary artery in patients with acute myocardial infarction [15–17]. In our previous publications regarding effects of a single intrathecal autologous stem/progenitor Lin^−^ cell infusion in ALS patients, we have in fact observed that this form of intervention may influence neurotrophin secretion, immune modulation in CNS environment and, moreover, affect articulatory functions [15, 17, 18]. Therefore, in this study, we have aimed to assess whether triple repeated administration of Lin^−^ cells could sustain these beneficial humoral effects and perhaps further prevent speech deterioration.

## 2. Materials and Methods

### 2.1. Subjects

The study was designed as a prospective, open-label, nonrandomized clinical trial in a single center for subjects with ALS. The trial (international number: NCT02193893) was approved by the Ethics Committee of the Pomeranian Medical University in Szczecin and conducted in accordance with the Declaration of Helsinki. Prior written informed consent was obtained from all of the subjects. Patients enrolled in the study met the following criteria:

1. Under 65 years of age
2. The diagnosis of a probable or certain sporadic ALS form based on the El Escorial Revised Criteria
3. Ability to express informed consent
4. Observation of the course of riluzole-controlled disease for 3 months preceding the use of cell therapy
5. Mild to moderate disability documented by satisfactory bulbar and spinal motor functions, i.e., minimum score 3 on the ALS Functional Rating Scale Revised (ALS-FRSr) for swallowing and 2 points for food preparation and walking
6. Forced vital capacity (FVC) result greater than or equal to 50%
7. Without cancer, signs of acute inflammation, diabetes, cardiovascular disease, chronic kidney, and liver disease, in euthyreosis, not receiving drugs that could affect bone marrow stem cell activity

The study enrolled 42 patients—eighteen females and twenty-four males—aged between 27 and 65 years (mean: 54.1 ± 8.94) with sporadic ALS according to the revisited El Escorial Revised Criteria [19]. The patients with predicted survival time of over 12 months established on the basis of the general and neurological condition were administered three times Lin^−^ SPCs at intervals of six weeks in the Department of Neurology of the Pomeranian Medical University in Szczecin.

The outcome measures were as follows:

1. Primary outcome measures: safety of repeated autologous bone marrow-derived Lin^−^ stem/progenitor cell infusion in enrolled patients
2. Secondary outcome measures: efficacy of repeated autologous bone marrow-derived Lin^−^ stem/progenitor cell infusion in enrolled patients

A 3-month period following the enrollment was dedicated to natural history observation, during which controlled administration of riluzole was continued. Patients over 65 were excluded from the study, as it had been previously demonstrated that cell growth of expanded in vitro stem cells is strictly related to the donor's age [20]. Patients with evidence of any concurrent illness or receiving any medications (including potentially other previously applied stem cell-based therapies) which might affect bone marrow were also excluded.

### 2.2. Speech Test: VHI Questionnaire

International research projects have demonstrated that the VHI questionnaire is a reliable tool for the subjective evaluation of speech in ALS patients and that the scores it provides are consistent. The 30-item VHI questionnaire comprising 3 subscales of 10 items each: the physical (P) items, functional (F) items, and emotional (E) items, is a recommended method of subjective assessment of the severity of speech disorders by ALS patients themselves. Cronbach's alpha for the entire cohort was 0.95, indicating high internal consistency of the 30 items. The VHI is a reliable and valid tool that can be recommended for ALS. The questionnaire is widely used throughout the world [21–26]. The subjects underwent the assessment on days 0, 7, and 28 following each Lin^−^ cell administration.

### 2.3. Speech Test: FDA

One of the most important objective tests for evaluating articulation organs is the FDA. The FDA is a standardized test which relies on a 9-point rating scale applied to a patient. It provides information based on the observation of oral structures, functions, and speech. The test evaluates the following functions: reflexes, respiration, tongue, lips, the soft palate, laryngeal, and intelligibility. A 5-point rating scale (a–e) is used for the assessment, where letter “a” represents norm, “b” mild severity, “c” moderate, “d” considerable severity, and “e” profound severity. FDA is also used to assess the severity of the articulatory organ disorders and to monitor the effects of treatment [27]. The test was conducted by a clinical speech therapist with 13 years of experience of treating neurological conditions, predominantly Parkinson's disease. The second edition of FDA utilizes the latest findings concerning motor speech disorders and their contribution to neurological diagnosis. It has good feasibility (missing data < 5%), a high reliability of the total score (0.94), an excellent interrater agreement for the total score (0.96), and moderate to large construct validity for 81% of its items [27]. The subjects underwent the assessment on days 0, 7, and 28 following each Lin^−^ cell administration.

### 2.4. Neurological Assessment

Assessment of disease progression was performed using two functional ALS scales—the Norris scale and ALS-FRSr—on day 0 (in the day of bone marrow collection and Lin^−^ cell administration) and 3, 5, 7, and 28 days after each cell application. Norris scale describes limb and bulbar functions. The limb scale evaluates 21 items and bulbar scale 13. Each item is rated in four ordinal categories [28]. ALS-FRSr is based on a questionnaire which allows the assessment of physical functions in activities of daily living, and it is the most widely used system for functional rating of ALS patients. It is divided into four clinical domains: (1) bulbar function, (2) fine motor function, (3) gross motor function, and (4) respiratory function. Each section includes 3 questions scored from 0 (loss of function) to 4 (normal function). Total score ranges from 0 to 48 [29].

### 2.5. Cells

After patients had signed informed consent, bone marrow (BM) was aspirated in local anaesthesia from the posterior iliac crest and immediately suspended in solution containing phosphate-buffered saline (PBS) (pH 7.2) and heparin (20 U/mL; Gibco, USA) to prevent clotting. BM was then centrifuged in density gradient medium (MP Biomedicals, Santa Ana, CA, USA). The obtained suspension of BM mononucleated cells (MNCs) was subjected to immunomagnetic separation procedures. Isolation procedures were performed according to the manufacturer's instructions, according to the Good Manufacturing Practice (GMP) conditions. Briefly, lineage-negative cells were isolated through negative selection using a MidiMACS separator (Miltenyi Biotec, Auburn, CA, USA) as described previously [14]. Before administration, the cells were maintained in 2 mL of sterile PBS.

### 2.6. Administration Procedure

The total Lin^−^ SPCs isolated at six-week intervals were each time slowly administered into the subarachnoid space via lumbar puncture (between L3/L4 or L4/L5). The number of administered Lin^−^ cells varied between different applications and patients. The mean number of Lin^−^ SPCs administered during the first, second, and third procedures, respectively: 6.47 × 106 ± 6.05, 6.99 × 106 ± 6.31, and 7.06 × 106 ± 7.22. After each injection, the patients were advised to maintain the supine position for at least 24 hours.

### 2.7. Multiplex Analysis of Neurotrophins and CRP Concentration

Concentrations of selected neurotrophins (brain-derived neurotrophic factor (BDNF), nerve growth factor (NGF), and neurotrophin 3 (NT-3)) and C-reactive protein (CRP) were assessed using multiplex Luminex technique (Luminex Corporation, Austin, TX, USA). Cerebrospinal fluid (CSF) samples were collected on the day of BM collection, before the intrathecal injection of the cells, and one week later. Plasma samples were collected on day 0, one week later, and 28 days after each procedure. Upon the analysis, samples were stored in -80°C. Assays were performed according to the manufacturer's protocol, as described previously [30].

### 2.8. qPCR miRNA Analysis

The expression of selected miRNAs (miR-1, miR-206, miR-133a, and miR-155) was analyzed using qPCR in CSF and plasma samples obtained during each procedure cycle in day 0 and day 7. Results of the expression were normalized to synthetic *Caenorhabditis elegans* miR-39 which was spiked in to each sample prior to the isolation of miRNA. The isolation was performed using NucleoSpin miRNA Plasma (Macherey-Nagel, Germany) according to the manufacturer's protocol from 400 *μ*L of bodily fluid. Further steps of analysis were performed as described previously [15].

### 2.9. Statistical Analysis

The statistical calculations were performed with STATISTICA 13 PL software. To test the distribution of collected data, the Shapiro-Wilk test was used. As the analyzed groups were relatively small, the analysis between given timepoints within each group was firstly performed using Friedman ANOVA and further with the Wilcoxon signed-rank test. Only these calculations which presented with significant results (*p* < 0.05) with both used tests are related to as statistically significant in this paper. The data between groups were assessed with the Mann–Whitney *U* test. Categorical data testing was performed with Pearson's chi-squared test. To investigate the correlations between different factors, Spearman's rank order coefficient was calculated. *p* value < 0.05 was considered statistically significant.

## 3. Results

**Figure 1:**
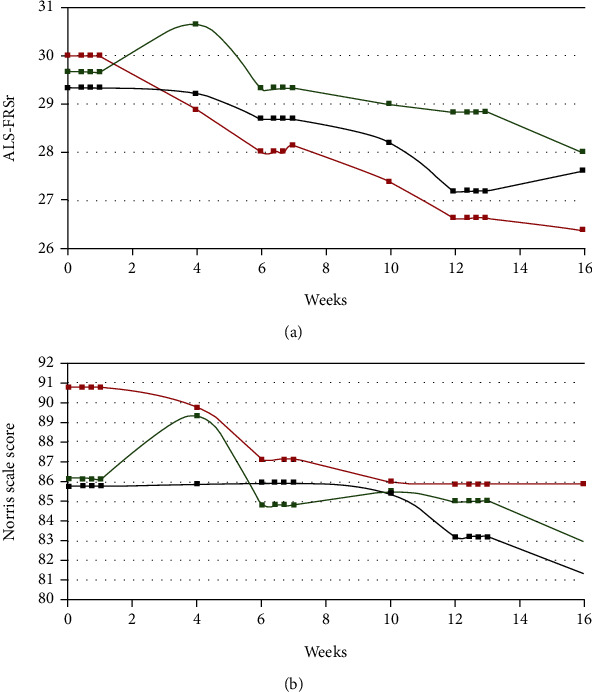
Functional assessment results with ALS-FRSr (a) and Norris scale (b) in three groups of sALS patients in time. Green—group I; black—group II; red—group III. The results are presented as the mean values. Squares represent the analyzed timepoints.

Overall, 42 sporadic ALS (sALS) patients were subjected to the triple procedure of autologous, intrathecal injection of Lin^−^ cells. As in case of single cells' injection, described in our previous publications, this time also we have not observed any acute or late adverse effects of the injection [15]. Retrospectively, patients were divided into three groups based on their objective articulatory functions (FDA assessment in day 0 before first cells' administration and in the last observation timepoint—28 days after third procedure): group I—patients whose functions improved (*n* = 6), group II—remained stable (*n* = 27), and group III—worsen during observation period (*n* = 9).  Table 1 shows the summary of anthropometric parameters and the number of administered Lin^−^ cells for each group separately. Additionally, the changes in the mean scores of ALS-FRSr and Norris scale assessments for all groups are presented in Figure 1. In general, we have observed a tendency that younger patients responded better in terms of speech functions improvement as the mean age of individuals included in group I was lower than those in the remaining two groups (statistically insignificant). In group I, the symptom duration was significantly longer than in the other groups and there was a tendency that patients from this group received the highest numbers of Lin^−^ cells, however statistically irrelevant. The analysis of Spearman's rank-order correlations revealed that the articulatory function improvement in response to cells' application was correlated with sex (*r*_*s*_ = 0.05, *p* < 0.05) as 5 out of 6 patients in group I were male. Out of 42 patients included in the study 10 (24%) presented with bulbar onset ALS. Correlation analysis has shown statistically significant correlation between patient's age and ALS duration (*r*_*s*_ = −0.43) and patient's age and the number of Lin^−^ cells administered in the first procedure (*r*_*s*_ = −0.42).

### 3.1. FDA Speech Assessment

**Figure 2:**
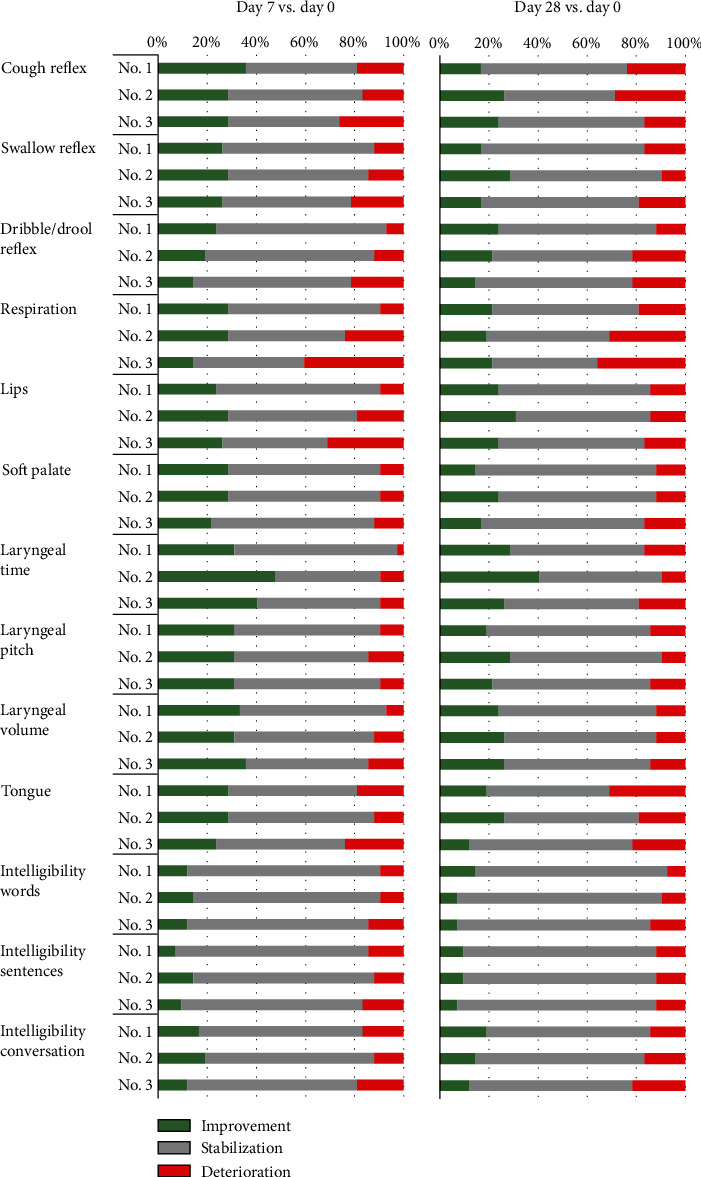
FDA scale assessment results after 7 and 28 days post each injection of Lin^−^ cells (No. 1, No. 2, No. 3) compared to the baseline values (day 0 of each procedure). Colors represent the percentage of patients (n =42) in which the analyzed parameter improved (green), remained stable (gray) or deteriorated (red).

The results of articulatory functions assessment utilizing FDA scale are presented in Figure 2. We have observed an improvement in cough reflex, swallow reflex, respiration, soft palate, laryngeal time, laryngeal pitch, laryngeal volume, and tongue parameters in more than 25% of patients after each consecutive administration of Lin^−^ cells. This effect was rather short-lived as it was noticeable predominantly until 7^th^ day and only exceptionally until the 28^th^ day after the procedure. Moreover, the statistical analysis revealed a significant, negative correlation between patient's age and FDA scale results one week after first administration procedure (*r*_*s*_ = −0.33), and between the overall FDA scale assessment (day 28 after the 3rd injection of Lin^−^ cells vs. day 0 before the first procedure) and the disease duration (*r*_*s*_ = −0.35).

### 3.2. VHI Speech Assessment

**Figure 3:**
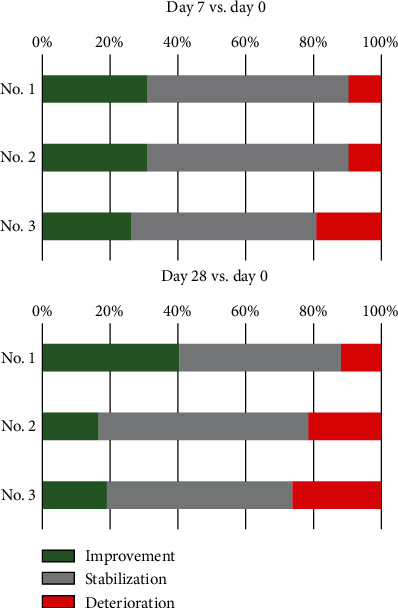
Subjective VHI questionnaire results after 7 and 28 days post each injection of Lin^−^ cells (No. 1, No. 2, and No. 3) compared to the baseline values (day 0 of each procedure). Colors represent the percentage of patients (*n* = 42) in which the analyzed parameter improved (green), remained stable (gray), or deteriorated (red).

The second scale—VHI—was used to assess subjective opinions of the patients on the quality of their articulatory functions after the procedure. Overall, the most beneficial effects, based on questionnaires obtained from patients, were observed after the first administration of the cells. Similar to FDA assessment, the most pronounced speech improvement was reported by patients on the 7^th^ day after each intervention. Unlike the FDA, subjective feeling of improved speech function was sustained until the 28^th^ day after the first procedure. Results are presented in Figure 3.

### 3.3. Concentrations of Neurotrophic Factors and CRP

**Figure 4:**
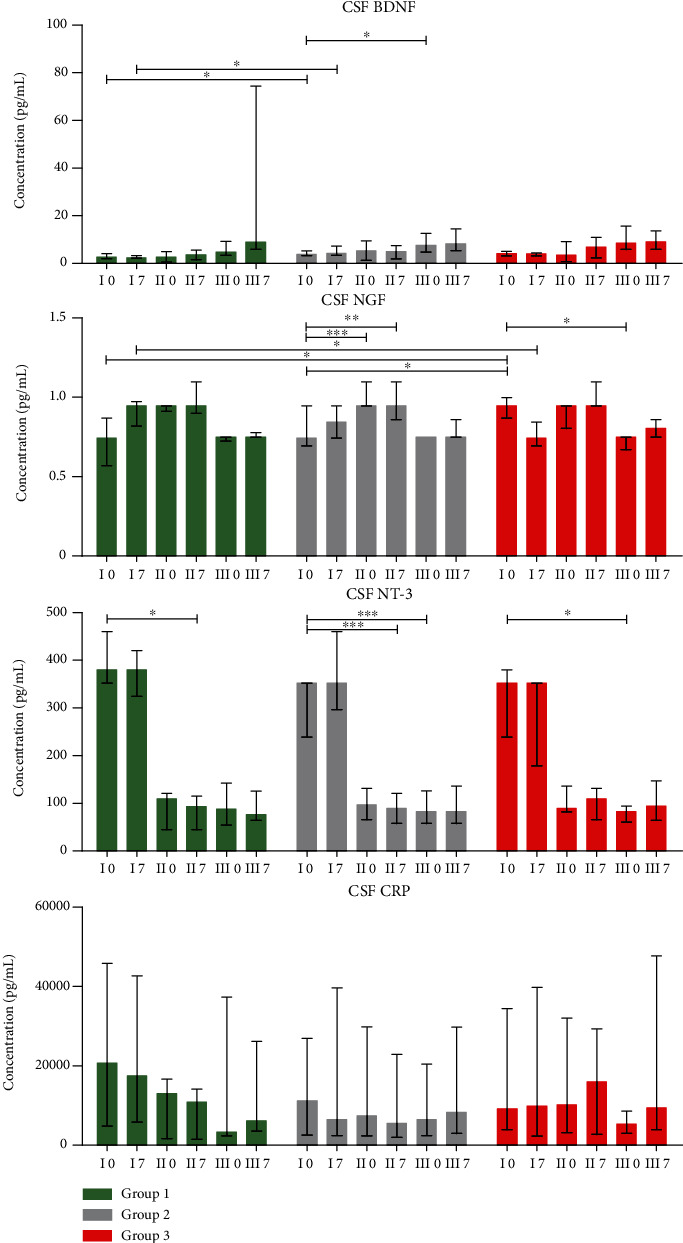
Concentrations of analyzed factors in CSF (pg/mL) of 42 sALS patients divided into three groups, based on the FDA scale results. The samples were collected in six timepoints presented on the *x*-axis. Roman numerals represent each Lin^−^ cell administration (I, II, and III), and Arabic numbers stand for the day of sample collection (0 and 7). Data are presented as the median values with an interquartile range. Level of significance: ^∗^*p* < 0.05, ^∗∗^*p* < 0.01, and ^∗∗∗^*p* < 0.001. BDNF: brain-derived neurotrophic factor; NGF: nerve growth factor; NT-3: neurotrophin 3; CRP: C-reactive protein.

**Figure 5:**
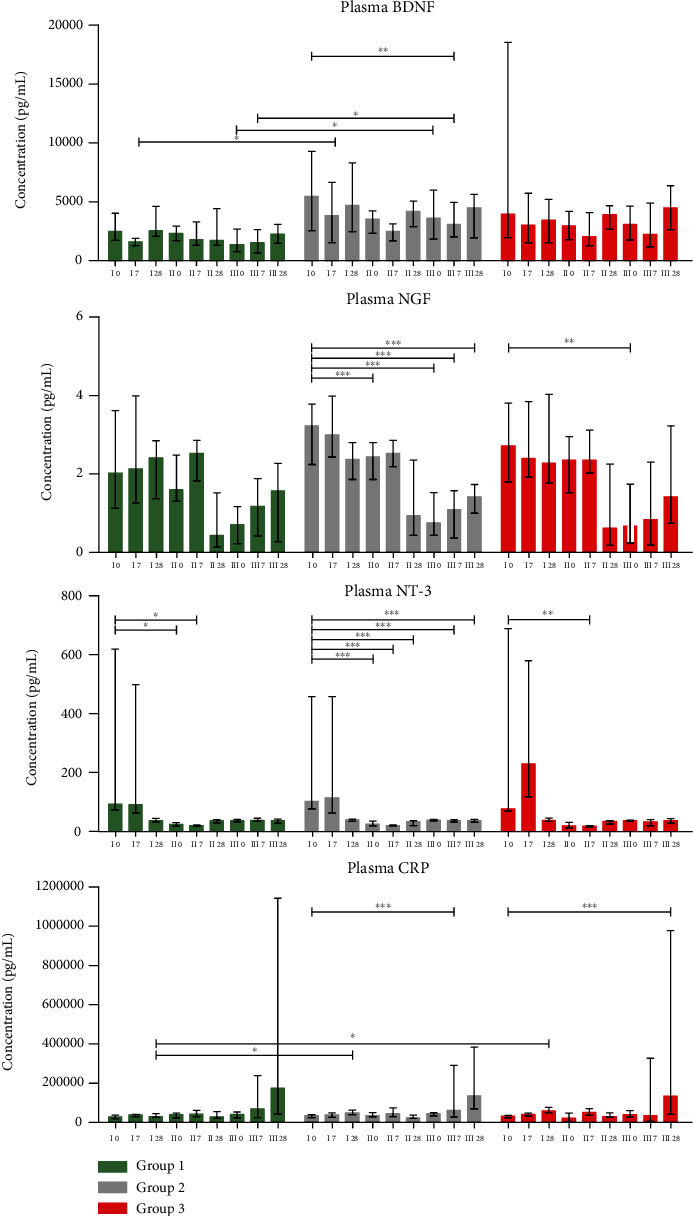
Concentrations of analyzed factors in plasma (pg/mL) of 42 sALS patients divided into three groups, based on the FDA scale results. The samples were collected in nine timepoints presented on the *x*-axis. Roman numerals represent each Lin^−^ cell administration (I, II, and III), and Arabic numbers stand for the day of sample collection (0, 7, and 28). Data are presented as the median values with an interquartile range. Level of significance: ^∗^*p* < 0.05, ^∗∗^*p* < 0.01, and ^∗∗∗^*p* < 0.001. BDNF: brain-derived neurotrophic factor; NGF: nerve growth factor; NT-3: neurotrophin 3; CRP: C-reactive protein.

The results of BDNF, NGF, NT-3, and CRP concentration measurements are presented in Figures 4 and 5, respectively, for CSF and plasma. Statistical analysis has shown significant, moderate positive correlation between the change of CRP concentration in CSF on the 28^th^ vs. 0 day after the first cells' injection and FDA scale result change between the second and first procedures (*r*_*s*_ = 0.49). Reversely, a moderate negative correlation was stated between the change in CSF CRP on day 0 for the second procedure vs. day 0 before the first injection of the cells and the change in VHI questionnaire results in the same timepoints (*r*_*s*_ = −0.49, *p* < 0.05).

### 3.4. miRNA Expression Analysis

**Figure 6:**
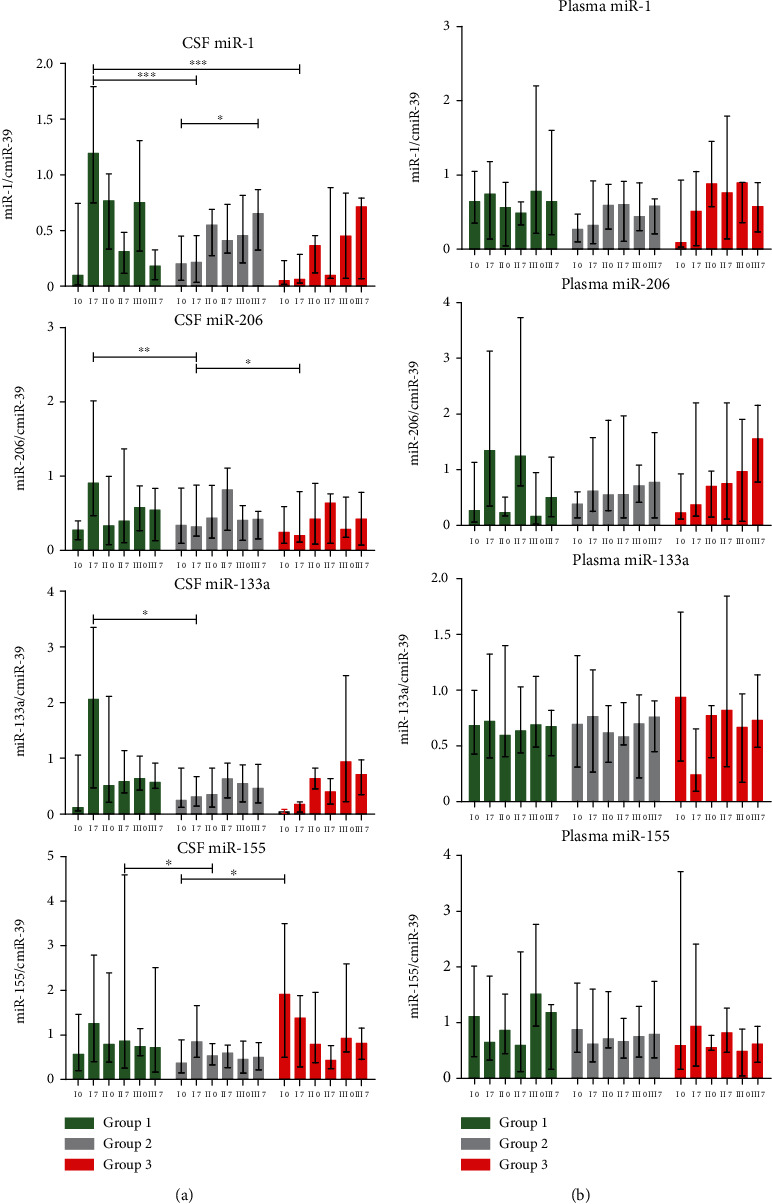
Results of qPCR miRNA expression analysis in (a) CSF and (b) plasma assessed in 42 sALS patients divided into three groups, based on the FDA scale results. The samples were collected in six timepoints presented on the *x*-axis. Roman numerals represent each Lin^−^ cell administration (I, II, and III), and Arabic numbers stand for the day of sample collection (0 and 7). Data are presented as the median values with an interquartile range. Level of significance: ^∗^*p* < 0.05, ^∗∗^*p* < 0.01, and ^∗∗∗^*p* < 0.001.

qPCR analysis of expression levels of selected miRNAs related to differentiation and regeneration of muscle cells (“myo-miRs”)—miR-1, miR-206, and miR-133a—and one immune response related, so-called “immune-miR”—miR-155—was performed in CSF and plasma samples before each cell's injection (day 0) and one week later (day 7). Results of the expression analysis in both bodily fluids are presented in Figure 6. We have observed a statistically significant increase in the expression of all analyzed “myo-miRs” in CSF from patients included in group I 7 days after the first administration of Lin^−^ cells. Expression levels of miR-1, miR-206, and miR-133a were additionally significantly higher in this timepoint when compared with corresponding samples collected from group II and, in the case of miR-1, group III. miR-155 expression was higher before the first administration procedure in patients from group III than in patients from the two remaining groups. We have not observed any significant changes in miRNA expression in plasma in assessed timepoints.

## 4. Discussion

Lineage-negative stem/progenitor cell population and utilization of its ability to secrete neurotrophic factors under stress, protein-poor conditions in approach to moderate the ongoing neurodegeneration in CNS of ALS patients, have been a focus of our research team over the past several years. However, in our previous works, we have presented data obtained from the observation of Lin^−^ cell influence on various aspects of the disease in sALS patients after a single administration procedure [15, 17, 18]. In previous experiments, we have confirmed that this cell's population constitutes a safe and feasible source of trophic support when administered intrathecally. Furthermore, we have found that single administration of cells exerts beneficial effects on articulatory functions, expressed particularly in better cough and dribble reflex as well as improved laryngeal time [18]. Therefore, in the presented study, we hoped to maintain this beneficial effect and to further explore the prognostic factors which correspond with better response to the therapy.

Our results have shown that triple intrathecal administration of Lin^−^ cells is safe as we have not observed any adverse reactions after the repeated injections of cell suspension. There were no significant differences between three analyzed groups of patients (I—with improvement of articulatory functions, II—those who remained stable when it comes to speech parameters, and III—individuals with articulatory functions deterioration) in the baseline functional results, obtained using ALS-FRSr and Norris scales. The speech improvement in patients from group I corresponded with better overall functional results, especially when assessed 28 days after the first procedure.

In patients included in groups II and III, we have observed a gradual decrease in functional condition; however, it must be mentioned that ALS is a quickly progressing disease and that the observation period presented in this study was relatively long (4 months) in relation to the estimated 3-5-year survival time in ALS [31].

Speech difficulties have been reported to occur in about 80-95% of ALS patients, and they are more severe in individuals with bulbar-onset [32]. The FDA scale analysis, which remains the most reliable indication of overall speech apparatus function, revealed short-lived improvement in laryngeal pitch and volume and moderate-termed improvement in laryngeal time in more than 25% of individuals enrolled in the study. This result is crucial as the abovementioned functions are vital for speech and it has been previously shown that the ability to speak correlates positively with the quality of life [11]. Similar improvement was reported regarding cough and swallow reflexes in over 25% of patients on the 7^th^ day after each procedure. This fact has beneficial implication for the prevention of aspiration and aspiration pneumonia-related death as the accumulation of saliva and obstruction in the airways may lead to respiratory failure which is the most common death cause in ALS patients [33]. Subjective, baseline speech function assessment (day 0 before first cells' administration) using VHI score revealed a significant difference between patients with spinal and bulbar-onset type (respectively, mean values of 16.3 vs. 77.3, *p* < 0.0001). The questionnaire results revealed also higher (worse) baseline VHI scores in women in comparison to male subjects (41.4 vs. 22.9 points, *p* = 0.07). Interestingly, the percentage of patients who reported speech improvement in subjective VHI questionnaire 28 days after first administration of cells was distinctively higher than the results obtained using FDA assessment. This result raises a question whether the observed effect was not partially caused by the patient's attitude towards the therapy and psychological aspects, which were not assessed in this study.

Neurotrophins have been shown to play an important role in all stages of neuronal system development and to regulate survival, regeneration, and neurotransmission in the mature nervous system [34]. Despite these pivotal functions, the use of their potential remains constrained due to their short half-life and lack of effective delivery rout into the CNS environment [35]. Therefore, we have aimed to utilize the selected population of SPCs—Lin^−^ cells as a vehicle for in situ NTs secretion. It was previously confirmed that transplantation of stem/progenitor cells in ALS mice provides neuroprotective effects by the production of trophic factors (including BDNF, NGF, and VEGF) that delay neurodegeneration and prolong survival in ALS mice [36, 37]. Our findings revealed an increase in concentrations of CSF BDNF on the 7^th^ day after each administration of cells. Interestingly, baseline levels of this factor were the lowest in patients included in the group with articulatory improvement (group I). We hypothesize that these low levels correlating with significantly longer disease duration in those individuals reflect sustained, slower neurodegeneration than in patients from remaining groups. Therefore, the lower levels of BDNF could be a consequence of its depletion in CNS. Similar tendencies were observed regarding concentration of NGF in CSF.

Inflammation plays a crucial role in the pathogenesis of ALS [38]. In our previous article, we have reported the effect of triple Lin^−^ cell administration on immunological pathway regulation [39]. Results obtained in presented study revealed an evident but not statistically significant gradual decrease in the level of CRP in CSF from patients with an improvement in articulatory functions. This protein has been previously described as a serum marker which correlates with the progression of ALS [40]. In fact, during the 16-week-long observation time, we have noticed an overall significant increase in concentrations of this protein in plasma samples of patients qualified to groups II and III. It is worth noting that four weeks after the first infusion of Lin^−^ cells, the CRP levels were significantly lower in ALS patients whose speech improved, suggesting a systemic suppression of immune response. Significantly lower levels of plasma CRP in patients from group I correlated with observed improvement in functional condition (ALS-FRSr and Norris scale) and better speech functions (laryngeal time and VHI results) 28 days after the first infusion of cells. Additionally, a significant difference between CRP concentration in plasma on the 28^th^ day post first infusion and day 0 (baseline) was confirmed between groups (I vs. II and I vs. III) in favour of a decline in group I.

In the last step of the analysis, we aimed at the assessment of miRNA expression. We have focused on four candidates which have been described previously as ones related to regulation of muscle cell regeneration (miR-1, miR-133a, and miR-206) and with immune processes (miR-155) [41]. MicroRNAs have been under investigation as a potential marker of numerous diseases and as factors which could help to predict the expected therapeutic approach efficacy [42, 43]. miR-206, apart from the role it plays in muscle cell regeneration, is also involved in the regeneration of neuromuscular synapses [44]. Changes in the expression profile of miR-1 and miR-133 have also been previously described in ALS studies [45, 46]. Expression levels of all analyzed “myo-miRs” increased in CSF of group I patients on the 7^th^ day after the first procedure. As such changes were not present in material collected from patients assigned to the other groups, it might suggest that the increased expression of those miRNAs exerts beneficial effects on the regulation of speech-related neural processes. miR-155 has been shown to exhibit both pro- and anti-inflammatory effects [47]. It acts, inter alia, via targeting NF-*κβ* signalling or by targeting calcium-regulated stable protein 1, what results in decreased stability of TNF-*α* mRNA [48, 49]. We have observed an increase in CSF levels of this molecule on the 7^th^ day after Lin^−^ cells' injection in patients from groups I and II which corresponds with a decrease of CRP concentration in this bodily fluid, further supporting the evidence of anti-inflammatory actions of miR-155. The assessment of circulating miRNAs in plasma did not reveal any significant changes, neither between analyzed groups nor in the consecutive timepoints. These findings stand in contradiction to previous reports which have suggested plasma miR-206 as a potential marker of the disease progression [50].

## 5. Conclusions

The parallel assessment of speech function and humoral response, presented in this study, allowed for a characterization of a subgroup of patients who could potentially benefit most from Lin^−^ cell application, regarding articulatory functions. We propose that male sex, longer disease duration, low baseline CSF levels of BDNF and NGF, and higher numbers of administered cells could indicate higher chances for speech improvement after the application of SPC therapy. These findings however need to be further investigated in larger cohort studies. Overall, the most significant results when it comes to both—modulation of humoral response and speech—were observed on the 7^th^ day after each cell infusion. Additionally, after the first procedure, the partial speech improvement, expressed in better VHI scores and laryngeal time according to FDA, was sustained until 4 weeks post Lin^−^ cell administration.

Although this study had some potential limitations resulting from lack of control, placebo group (due to ethical concerns), relatively long period of time between subsequent clinical assessments, and short follow-up caused by patients' deteriorating condition and geographical distance from research center, it certainly sheds a new light on the influence of SPC therapy on articulatory functions in ALS patients—a research area which to date has been very sparsely investigated.

## Figures and Tables

**Table 1:** Clinical characteristic of the study groups.

Characteristic	Group I (*n* = 6)	Group II (*n* = 27)	Group III (*n* = 9)	*p* value		
				I vs. II	I vs. III	II vs. III
Age (mean ± SD, years)	52.7 ± 10.7	53.6 ± 9.7	56.7 ± 4.6	0.91^a^	0.69^a^	0.77^a^
Sex (male/female)	5/1	16/11	3/6	**0.05** ^b^		
Disease onset (bulbar/limb)	0/6	7/20	3/6	0.30^c^		
Symptom duration (mean ± SD, months)	62.3 ± 41.8	23.8 ± 18.0	20.7 ± 14.3	**0.01** ^a^	**0.03** ^a^	0.70^a^
Number of Lin^−^ cells administered (mean ± SD)						
No. 1	(8.12 ± 8.57) × 106	(6.92 ± 6.25) × 106	(4.01 ± 2.38) × 106	0.60^a^	0.95^a^	0.19^a^
No. 2	(8.97 ± 8.34) × 106	(6.78 ± 6.0) × 106	(6.31 ± 6.31) × 106	0.48^a^	0.33^a^	0.69^a^
No. 3	(12.57 ± 15.27) × 106	(6.48 ± 4.68) × 106	(5.14 ± 4.73) × 106	0.60^a^	0.33^a^	0.28^a^
Baseline (day 0) ALS-FRSr score (mean ± SD)	29.7 ± 5.1	29.3 ± 5.1	30.0 ± 5.2	0.95^a^	0.95^a^	0.80^a^
Baseline (day 0) Norris scale score (mean ± SD)	86.2 ± 12.6	85.7 ± 15.3	90.8 ± 15.7	0.80^a^	0.69^a^	0.52^a^

^a^Mann–Whitney *U* test. ^b^ Spearman's rank correlation coefficient. ^c^Pearson's chi-squared test. *p* < 0.05 is highlighted in bold font.

## Data Availability

Data available are on request.
